# Populational influence on cephalometric landmark identification: performance of two AI-driven software programs in Brazilian and Korean images

**DOI:** 10.1186/s12903-025-06807-4

**Published:** 2025-10-10

**Authors:** Thaisa Pinheiro Silva, Giovanna Sachs Puntigam, Maria Fernanda Silva Andrade-Bortoletto, Wilton Mitsunari Takeshita, Christiano Oliveira-Santos, Deborah Queiroz Freitas

**Affiliations:** 1 Department of Oral Diagnosis, Division of Oral Radiology, Piracicaba Dental School, University of Campinas (UNICAMP), Piracicaba, Sao Paulo, 13414-903 Brazil; 2 Department of Oral Diagnosis, Division of Oral Radiology, , School of Dentistry of Araçatuba, Paulista State University (UNESP), Araçatuba, Sao Paulo, 16015-050 Brazil; 3 Department of Diagnosis and Oral Health, University of Louisville School of Dentistry, Louisville, KY U.S.A.

**Keywords:** Artificial intelligence, Cephalometry, Image processing, Computer-Assisted, Radiography, dental

## Abstract

**Objective:**

To assess the performance of cephalometric landmark identification performed by two AI-driven software programs in images from different populations (Brazilian and Korean).

**Methods:**

Sixty lateral cephalometric radiographs (30 Brazilian and 30 Korean) were analyzed. The Brazilian images were acquired using the Orthophos XG 5/Ceph device, while the Korean images were obtained from the International Symposium on Biomedical Imaging 2015 database. Images of patients with permanent dentition were included, excluding those with poor head positioning or severe craniofacial deformities. Twenty cephalometric landmarks were identified by two examiners used as the reference standard. Two AI-driven software programs, CefBot™ (Brazil) and WebCeph™ (Korea), automatically identified the same landmarks. Coordinate values for each landmark were measured using ImageJ, and the data were analyzed with Analysis of Variance and Dunnett’s post-hoc test.

**Results:**

The Brazilian software showed high accuracy in identifying landmarks on Brazilian images (90%) but was less precise on Korean images (80%), with significant discrepancies in the Glabella, Menton L, Basion, and Orbitale landmarks. Similarly, the Korean software had a higher accuracy in its own population (95%) than in another population (85%), with notable inaccuracies in the Menton L, Basion, and Porion landmarks.

**Conclusion:**

Discrepancies in the identification of specific landmarks, such as Glabella and Menton L, suggest that the accuracy of the software may be influenced by the training process itself and by the population origin of the training data.

## Background

Artificial Intelligence (AI) has been substantially contributing to technological advancement in various fields, enabling software to mimic human behavior, especially in tasks involving pattern recognition [1]. In this context, Dental Radiology is considered the most promising area in Dentistry for the application of AI due to its digital nature, which can be easily converted into computational language [2]. 

The training of an AI software for image detection/classification involves various stages. The training phase is the step in which the algorithm receives sufficient information to recognize patterns and learn the behavior it should replicate later. Training should be conducted with minimal errors and the greatest possible variation of data. In cases where, after training, the software cannot reproduce its performance for data different from what it was trained on, overfitting occurs, in such cases, training must be adjusted, or the network should be retrained [3, 4]. 

Tasks such as the identification of cephalometric landmarks are already performed by different AI models. The accuracy in this task has evolved over time, which is crucial to enable its application in clinical practice. Currently, good performance of some software is reported in the literature [5–10]. 

CefBot™ (RadioMemory, MG, Brazil) is a Brazilian AI software with excellent accuracy in identifying cephalometric landmarks [11–13]. However, the previous studies have used images of Brazilian patients, limiting the results to this specific population. On the other hand, WebCeph™ (AssembleCicle, Korea) is a Korean AI software previously reported in the literature as inaccurate for identifying cephalometric landmarks [13, 14]. The radiographs used in the studies were from an unknown population [14] and from Brazilian skulls [13]which could explain the inaccurate results for its performance.

If a software was trained on a population different from the one it was evaluated on, this can lead to discrepancies in its performance. Differences in facial and cranial characteristics among populations can impact the model’s ability to effectively generalize to new cases. Therefore, considering that the images selected for the algorithm training can strongly influence the final performance of the AI software, the aim of this study was to assess the performance of cephalometric landmark identification performed by two AI-driven software programs in images from different population origins (Brazilian and Korean) based on the comparison of the coordinates of each point with human identification.

## Methods

This study was registered and approved by the appropriate institutional review board under the protocol number CAAE 70764623.0.0000.5418 and was carried out according to the Declaration of Helsinki. All Brazilian individuals assigned an informed consent for their radiographs to be included in the study. Consent could not be obtained for the radiographs of the Korean individuals because the authors did not have access to them. The radiographs were collected from an open-access bank. By using an open and free-access bank, we assumed that those responsible for the bank followed the ethical principles of their country.

### Sample preparation

Thirty lateral cephalometric radiographs of Brazilian individuals were selected from the archives of Oral Radiology Department, acquired with the Orthophos XG 5/Ceph device (Dentsply Sirona, York, PA) and 30 lateral cephalometric radiographs of Korean individuals from the open and free-access files available at the International Symposium on Biomedical Imaging, held in Korea (2015): From Nano to Macro event (https://isbi2019.signalprocessingsociety.org/biomedicalimaging.org/2015/program/isbi-challenges/index.html). Lateral cephalometric radiographs of individuals with permanent dentition were included in the study. Images were excluded if they demonstrated poor head positioning in the cephalostat, apparent image distortions, or clear evidence of craniofacial deformities and asymmetries. These exclusions were determined through visual inspection of the radiographs by two experienced radiologists working in consensus and comprised features such as marked skeletal disproportions, asymmetrical jaw contours, or evident deviations from standard anatomical alignment. As this was a retrospective study, no additional clinical information or anamnesis forms were available for evaluation. The images were extracted from the system in JPEG format, with maximum dimensions of 520 × 584 pixels.

Twenty cephalometric landmarks, corresponding to anatomical structures located in both osseous and soft tissue, were selected for the study: Basion (Ba), Posterior Nasal Spine (PNS), Anterior Nasal Spine (ANS), Menton (Me), Nasion (Na), Orbitale (Or), Pogonion (Pog), Porion (Po), Subspinale (A), Supramentale (B), Nasion Line (Na’), Pogonion Line (Pog’), Supramentale Line (B’), Lower Lip (LL), Upper Lip (UL), Subspinale Line (A’), Subnasale (Sn), Menton Line (Me’), Glabella (G), and Nasal Tip (N).

### Reference standard and cephalometric identification

Two examiners, previously trained in cephalometric tracing and blinded to the population origin of the images, independently identified the twenty cephalometric landmarks on the 60 images using the RadioCef Studio 3 software (RadioMemory, MG, Brazil) without the AI module. For this purpose, the images were uploaded to its online platform with dimensions of 520 × 584 pixels, allowing manual adjustment of brightness, contrast, and zoom. Assessments were conducted using a 13-inch MacBook Air (Apple, Intel Core i5 processor, 8GB memory, 256GB storage, Intel HD Graphics 6000, and LED display). Once the identification of cephalometric landmarks was completed, the images were downloaded and saved in JPEG format. After the examiners performed the evaluation of 10 images, the intra-examiner reproducibility was calculated to check their consistency. Since the Intraclass Correlation Coefficient (ICC) showed excellent agreement (ICC = 0.999), the examiners continued the evaluation of the images.

**Fig. 1:**
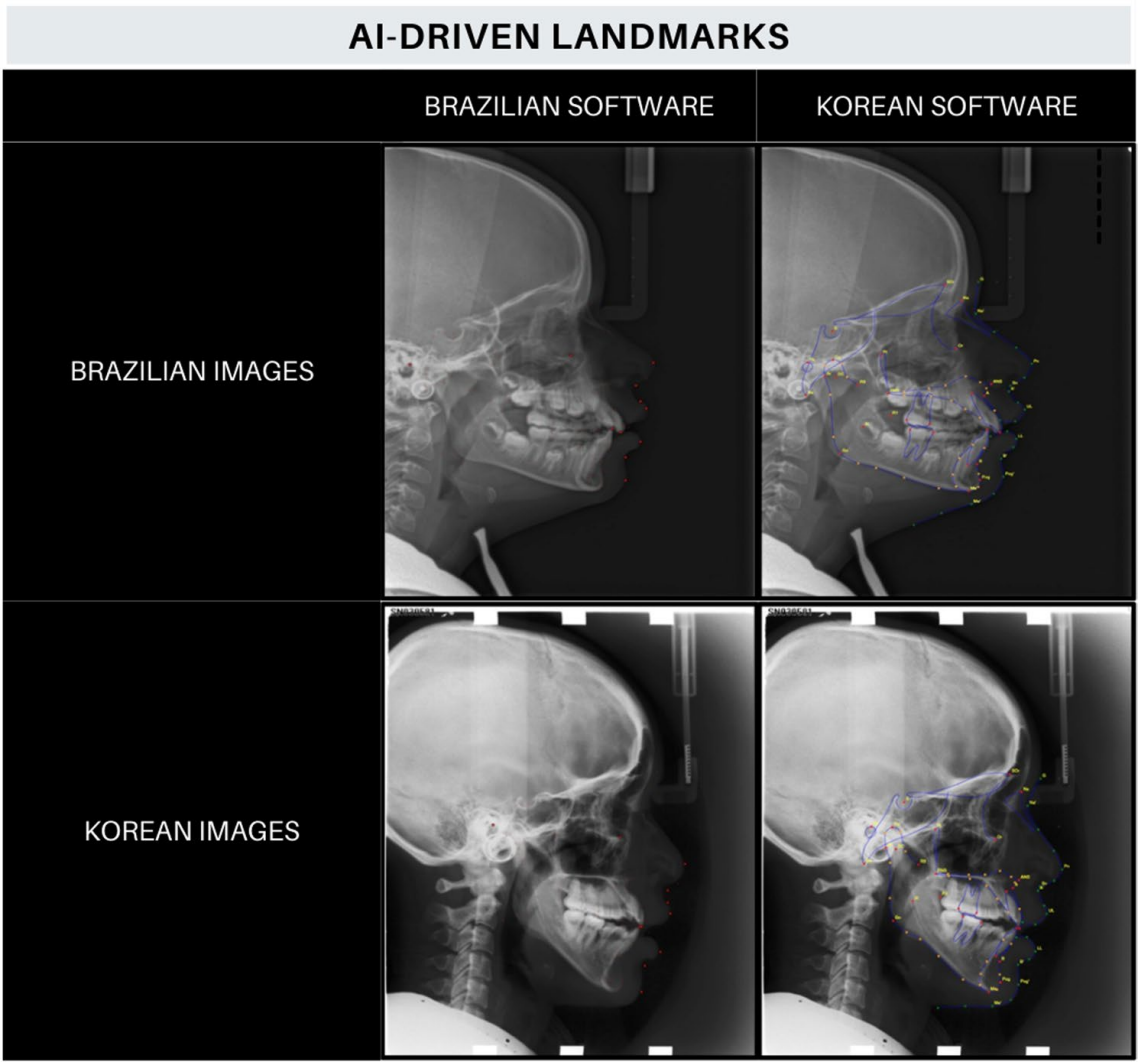
Lateral cephalometric radiographs from the sample images of Brazilian and Korean individuals after automated cephalometric identification performed by the AI-driven software programs CefBot™ and WebCeph™

Two AI-driven software programs, CefBot™ (RadioMemory, MG, Brazil), and WebCeph™ (AssembleCicle, Korea), which will be called Brazilian and Korean software respectively, were used to automatically identify twenty cephalometric landmarks in images of Brazilian and Korean individuals. For this purpose, 60 images from both population (30 of Brazilian and 30 of Korean individuals) were uploaded to the online platform of the Brazilian software by an examiner different from those who evaluated the images. Upon activating its AI module, the cephalometric landmarks in question were identified. The properly identified images were saved in JPEG format, with a size of 520 × 584 pixels. The same 60 images were uploaded to the online system of the Korean software, where, upon activating the AI tool, the cephalometric landmarks were identified, generating final images in JPEG format with a size of 520 × 584 pixels. Figure 1 presents lateral cephalometric radiographs from two population origins (Brazilian and Korean) after automatic identification of cephalometric landmarks performed by the two AI-driven software programs.

**Fig. 2:**
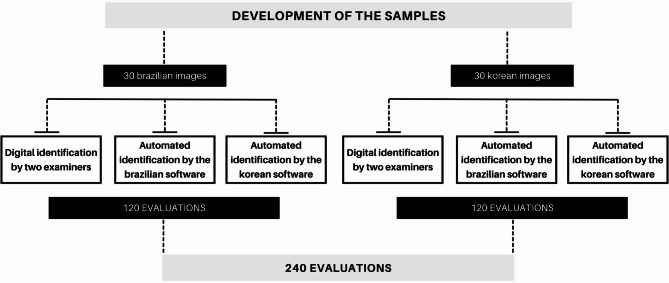
Development of the sample. Coordinates were extracted from a total of 240 images (60 images x 2 examiners + 60 images x 2 AI-driven software)

Image J software (National Institutes of Health, Bethesda, MD, USA) was used to assess the coordinate values for each point of all 240 images (60 images x 2 examiners + 60 images x 2 AI-driven software) (Fig. 2). The values of the X (abscissa) and Y (ordinate) coordinates as displayed by positioning the cursor on the cephalometric point were recorded.

Thirty days after the first assessment, 30% of the images were randomly selected and submitted to a new cephalometric identification by the two software programs, following the same protocols.

### Statistical analysis

The data were analyzed in SPSS software version 23 (IBM Inc., Chicago, IL, USA), with a significance level of 5% and an analysis power of 90%, which was post-calculated considering the minimal difference among the groups, the mean standard deviation and the number of repetitions per group.

The intra-examiner agreement was calculated for the entire data and was also excellent (0.999 for both x-axis and y-axis). Therefore, the means of results from the examiners were used as reference standard.

The data was tested for normality by the Shapiro-Wilk test. Analysis of Variance (two-way ANOVA) with Dunnett’s post-hoc test compared the values found by the AI-driven software programs and the reference standard. For this comparison, the raw data collected on ImageJ software was used. However, for easier interpretation, Table 1 presents the mean differences between the reference standard and the landmark positions identified by the software programs.

**Table 1:** Mean and standard deviation (SD) difference between the coordinates (in millimeters) determined by the examiners and the AI-driven software programs for the X and Y axes, according to the points and different population origins

Landmark	Software program	X^a^					Y^b^		
		Brazilian image	SD	Korean image	SD	Brazilian image	SD	Korean image	SD
Basion	Brazilian	−0.25	0.13	−1.88*	0.12	−0.05	0.38	−1.90	0.15
	Korean	−0.37*	0.13	−0.67	0.12	0.68	0.38	−1.19	0.15
ANS	Brazilian	−0.05	0.51	−0.65	0.20	−0.01	0.41	0.14	0.25
	Korean	0.72	0.51	−1.83	0.20	0.66	0.41	−0.92	0.25
PNS	Brazilian	−0.15	0.28	−1.80	0.15	0.01	0.38	0.61	0.18
	Korean	0.26	0.28	−1.32	0.15	0.63	0.38	−0.97	0.18
Glabella	Brazilian	13.30*	0.53	92.23*	0.15	15.65*	0.60	88.11*	0.20
	Korean	0.94	0.53	0.22	0.15	1.24	0.60	4.84*	0.20
Lower Lip	Brazilian	0.06	0.58	0.32	0.25	0.02	0.32	0.03	0.42
	Korean	0.97	0.58	0.07	0.25	0.35	0.32	−3.15	0.42
Upper Lip	Brazilian	0.13	0.58	−0.89	0.28	−0.01	0.36	−0.28	0.35
	Korean	1.00	0.58	−1.12	0.28	0.63	0.36	−1.40	0.35
Menton	Brazilian	−0.02	0.49	−0.35	0.30	−0.03	0.29	−0.29	0.56
	Korean	1.16	0.49	1.89	0.30	0.35	0.29	−3.55	0.56
Menton L	Brazilian	13.08*	0.49	87.76*	0.24	3.21*	0.24	17.04*	0.59
	Korean	2.06*	0.49	6.86*	0.24	0.65*	0.24	−1.81*	0.59
Nasion	Brazilian	−0.02	0.49	0.09	0.18	−0.02	0.58	−0.44	0.20
	Korean	0.93	0.49	0.21	0.18	1.16	0.58	0.61	0.20
Nasion L	Brazilian	−0.02	0.52	0.30	0.17	0.01	0.55	0.01	0.19
	Korean	1.01	0.52	1.03	0.17	1.53*	0.55	5.10*	0.19
Orbitale	Brazilian	−0.42	0.43	−2.17*	0.14	−0.08	0.48	−0.48	0.15
	Korean	0.24	0.43	−1.69	0.14	0.67	0.48	0.17	0.15
Pogonion	Brazilian	0.01	0.52	−0.38	0.30	0.05	0.29	0.82	0.53
	Korean	0.79	0.52	−0.35	0.30	0.38	0.29	−2.38	0.53
Pogonion L	Brazilian	0.08	0.57	0.31	0.29	0.25	0.29	1.15	0.53
	Korean	0.96	0.57	0.21	0.29	0.29	0.29	−3.07	0.53
Nasal Tip	Brazilian	0.28	0.64	0.23	0.18	−0.09	0.44	−0.54	0.29
	Korean	1.10	0.64	0.12	0.18	0.69	0.44	−0.58	0.29
Porion	Brazilian	−0.30	0.13	−1.14	0.09	−0.13	0.45	−0.84	0.10
	Korean	−0.37*	0.13	−0.82	0.09	0.92	0.45	−0.70	0.10
Sella	Brazilian	−0.09	0.19	−0.14	0.13	0.09	0.52	0.37	0.13
	Korean	0.16	0.19	0.21	0.13	0.88	0.52	0.64	0.13
Subespinale	Brazilian	0.12	0.50	0.57	0.19	−0.01	0.39	0.49	0.27
	Korean	0.79	0.50	−1.36	0.19	0.83	0.39	−0.31	0.27
Subnasale	Brazilian	0.00	0.57	−0.95	0.17	0.04	0.40	−0.44	0.28
	Korean	0.94	0.57	−0.15	0.17	0.59	0.40	−2.12	0.28
Supramenton	Brazilian	0.02	0.50	−0.02	0.27	−0.26	0.30	−1.17	0.46
	Korean	0.84	0.50	0.02	0.27	0.28	0.30	−3.33	0.46
Supramenton L	Brazilian	−0.07	0.55	−0.44	0.27	−0.07	0.30	0.05	0.48
	Korean	0.89	0.55	0.08	0.27	0.41	0.30	−3.40	0.48

^a^Measurements of Brazilians and Koreans differed at all points for the X-axis, regardless of the software used

^b^Measurements of Brazilians and Koreans did not differ on the Y-axis

*Significant difference, according to ANOVA and Tukey’s post-hoc test (*P* < 0.05)

For the x-axis, a negative mean difference indicates that the landmark identified by the software is located to the right of the corresponding landmark in the reference standard. Conversely, a positive mean difference indicates the landmark is located to the left of the reference landmark.

For the y-axis, a negative mean difference indicates that the landmark identified by the software is positioned above the corresponding landmark in the reference standard, whereas a positive mean difference means it is located below the reference landmark.

The reproducibility between the first and second identification performed by the AI-driven software programs was analyzed using ICC. The pixel coordinate values were converted to millimeters by applying the rule of three (1 pixel = 0.04 mm for Brazilian images, and 1 pixel = 0.23 pixels for Korean images).

## Results

Table 1 shows the mean differences between the coordinates determined by the examiners and the AI-driven software programs for the x- and y- axes. For the x-axis, the Brazilian software demonstrated good performance for images of Brazilian patients for most of the landmarks, showing no significant difference with the reference standard in general (*p* > 0.05). Exception to this was observed for the landmarks Glabella (13.30 mm) and Menton L (13.08 mm), which presented significant difference when compared to the reference standard (*p* < 0.05). Thus, an accurate identification rate of 90% for the cephalometric landmarks was observed. For the Korean images, in addition to the previously mentioned landmarks, the Basion and Orbitale landmarks presented differences (1.88 mm and 2.17 mm, respectively), resulting in in 80% of the landmarks addressed that didn’t differ significantly from reference standard.

**Fig. 3:**
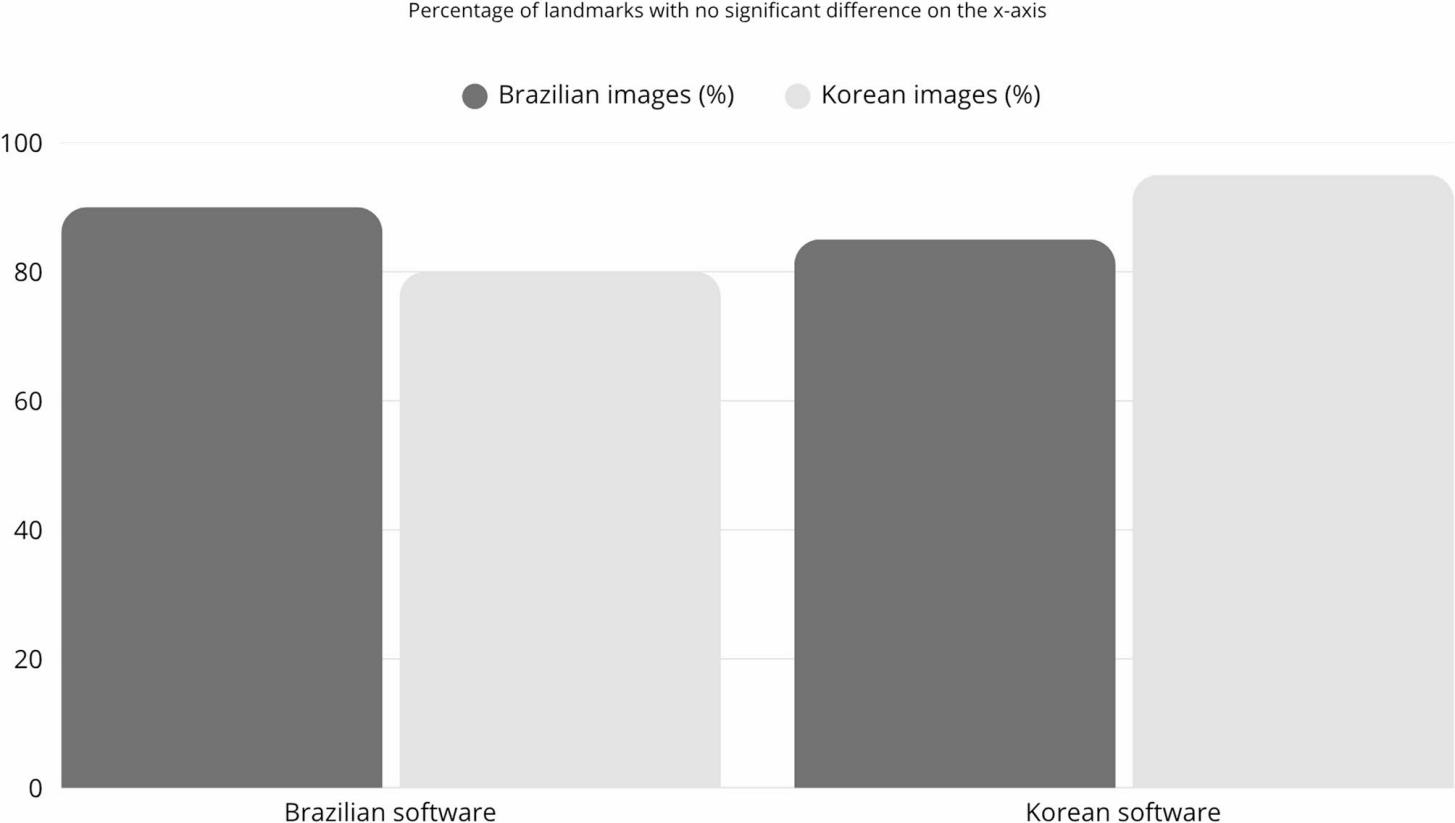
Bar chart depicting the percentage of landmarks identified by the two AI-driven software programs that showed no significant difference when compared to the reference standard for the x-axis

The Korean software, for this same axis, showed significant differences for the Basion, Menton L, and Porion landmarks (0.37 mm, 2.06 mm, and 0.37 mm, respectively) for the Brazilian images, achieving 85% of correct identification of the addressed landmarks. For the Korean images, it was observed that only Menton L point (6.86 mm) showed a significant difference, achieving an identification rate of 95% of the addressed landmarks. Figure 3 illustrates the percentage of cephalometric landmarks that did not differ significantly from the reference standard for each software in relation to different population origins for the x-axis.

For the Y-axis, the Brazilian software exhibited significant differences for the Glabella and Menton L landmarks (15.65 mm and 3.21 mm, respectively) in Brazilian images, achieving 90% success rate in identifying the addressed landmarks. For the Korean images, the Glabella (88.11 mm) and Menton L (17.04 mm) landmarks also showed significant differences along this axis, with the software achieving 90% success rate in the cephalometric landmarks.

**Fig. 4:**
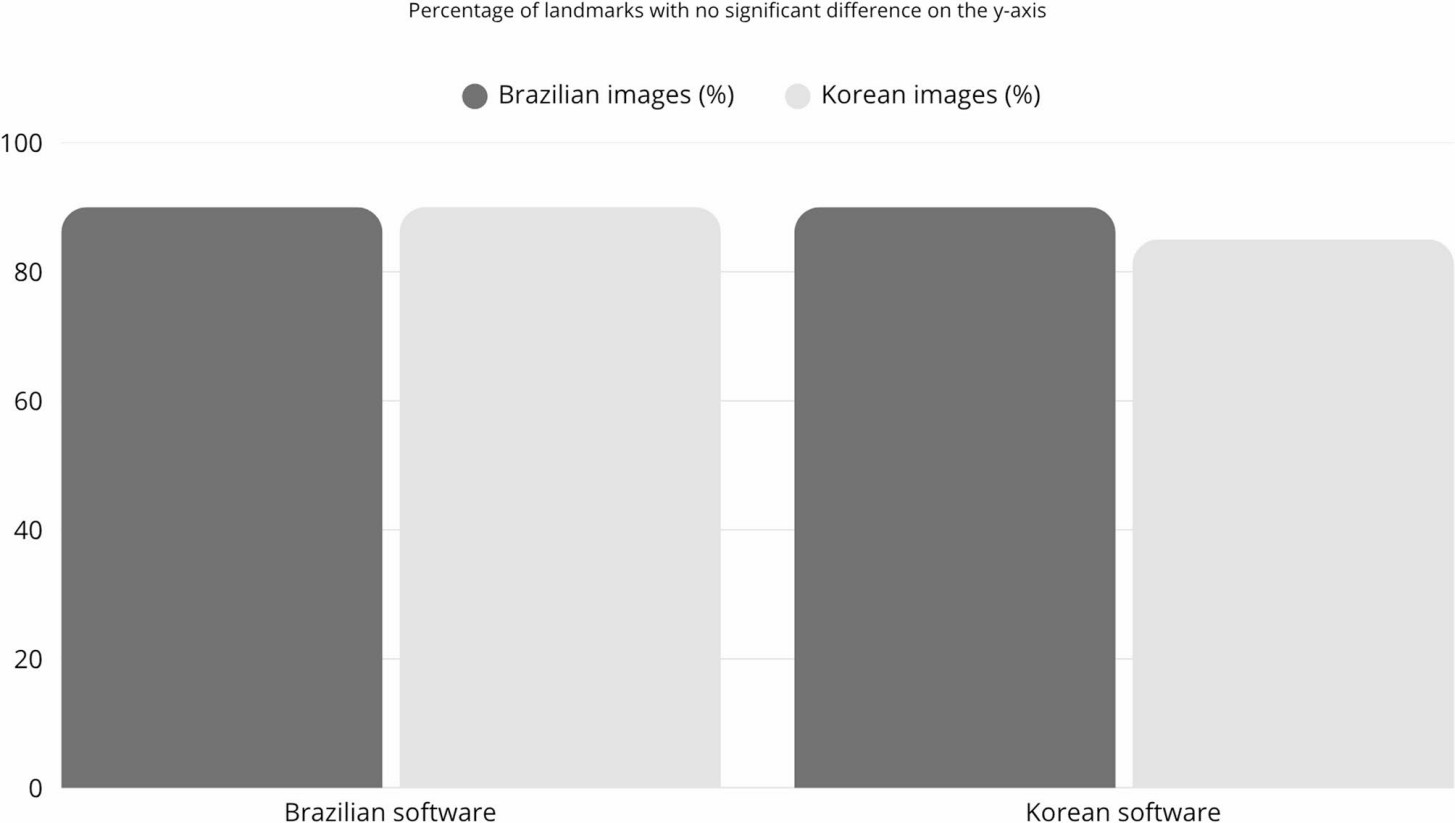
Bar chart depicting the percentage of landmarks identified by the two AI-driven software programs that showed no statistical difference when compared to the reference standard for the y-axis

Regarding the Y-axis, the Korean software showed significant differences for the Menton L and Nasion L landmarks (0.65 mm and 1.53 mm, respectively) for the Brazilian images, indicating a 90% correctness in the identification of the cephalometric landmarks in this study. For the Korean images, the Glabella (4.84 mm), Menton L (1.81 mm), and Nasion L (5.10 mm) landmarks showed significant differences, achieving correctness in 85% of the studied landmarks. Figure 4 illustrates the correctness percentage of each software in relation to different population origins for the specified axis. ICC showed excellent agreement for both programs in both axes (ICC = 1.0).

## Discussion

This study is pioneering in its evaluation of the performance of two AI-driven software programs for cephalometric landmark identification across different populations, specifically Brazilian and Korean images. The Brazilian software demonstrated great performance within its native population, but encountered challenges with certain landmarks, such as Glabella and Menton L in both populations. Similarly, while the Korean software maintained consistent performance across both Brazilian and Korean images, some discrepancies were noted in landmark identification. Given the novelty of this comparative approach, it was challenging to find existing studies that directly addressed the performance of AI-driven cephalometric analysis based on different population origins.

Although the Brazilian and Korean software demonstrated comparable overall performance in identifying most cephalometric landmarks, certain discrepancies, particularly in the localization of the Glabella and Menton L points, may hold clinical importance. In contexts requiring high diagnostic precision, such as orthodontic planning, orthognathic surgery, or craniofacial growth assessment, even subtle landmark identification errors can lead to variations in treatment planning. These findings suggest that AI-driven diagnostic tools may benefit from population-specific calibration to ensure reliable performance across diverse demographic groups.

The Brazilian software demonstrated overall good performance in Brazilian images, achieving successful rate in 90% of the landmarks. However, some discrepancies suggest potential variations in landmark identification performance. That result agrees with preview ex-vivo study that evaluated the accuracy of the same software and showed a good performance for landmark identification [13]. In Korean images, the general successful rate decreased to 85% of the landmarks, indicating potential challenges in cross-population application.

In general, the Korean software showed a great performance for both Brazilian and Korean images, but there was also a decrease in the accuracy when another population was evaluated. This behavior was not observed for specific landmarks (Basion, Menton L, Porion, and Nasio L) in Brazilian images, achieving yet no significant difference in 87.5% of the landmarks in both axes. In Korean images, the software excelled with correct identification in 92.5% of the landmarks, indicating robust performance in its native population, presenting significant difference with the reference standard only for Glabella, Menton L, and Nasio L landmarks. Different from the present result, previous studies have indicated low performance and inconsistency for this same Korean software in identifying cephalometric landmarks [13, 14]. This may be due to the different assessed population and the influence of new training to improve it functionalities.

When comparing the performance of the two software, they showed a slightly better performance when analyzing the images of their own population. However, the difference was small, and both had difficulty in identifying some landmarks, regardless of the population. This suggests that the observed limitations may be due to the training process itself, rather than the origin of the images, and indicates a need for further training regarding the anatomical localization of some cephalometric landmarks. On the other hand, it is important to highlight that their performance varied more in identifying landmarks in the x-axis, when the performance was more related to the population, than in the y-axis, which showed was more consistent results, regardless of the population, as it can be seen in Figs. 3 and 4. Considering that the ANOVA showed a significant difference between the pattern of the population only on the x-axis, it can be inferred that the origin of the images has some influence on their performance, because of the pattern of the images used in the training process.

It is important to note that the Brazilian software showed significant differences for the same landmarks for Brazilian and Korean images (Glabella and Menton L). Again, this suggests a potential difficulty in identifying these specific landmarks, which may not be directly related to the software’s training on different populations. For instance, the Glabella point has previously been reported as unidentifiable by the Brazilian software in earlier study [11]. 

It is important to highlight that most of the soft tissue landmarks showed good performance for both software and for both population, except for the Glabella landmark for the Brazilian software in both population, for Menton L landmark for both software and population, Nasio_L landmark for the Korean software in both populations. For the other soft tissue landmarks (Nasion L, Pogonion L, Supramentale L, Lower Lip, Upper Lip, Subspinale L, Subnasale, and Nasal Tip) the accuracy was high. That is in accordance with the result of a preview study in which the localization of cephalometric landmarks using a different AI-based software showed high consistency for the soft tissue ones [15]. 

Ribas-Sabartés et al. (2024) [16] conducted a systematic review demonstrating that different AI algorithms, such as convolutional neural networks (CNN), machine learning (ML), and artificial neural networks (ANN), exhibit variable performance in landmark detection depending on their architecture and training phases. These findings suggest that the observed discrepancies in our study could be partially attributed to differences in the AI models themselves, beyond population-related factors.

The study’s findings underscore the importance of considering population-specific variations in cephalometric landmark identification when using AI-driven software. These insights are crucial for improving diagnostic precision and treatment planning in orthodontics and maxillofacial surgery, where accurate cephalometric analyses are paramount. Limitations such as sample size and specific software capabilities should be considered. Future studies could explore additional demographic factors influencing landmark identification accuracy and validate findings with larger, more diverse datasets. Incorporating new algorithms and updating techniques to refine software algorithms based on regional variations could further enhance accuracy and clinical utility.

## Conclusions

The Brazilian and Korean software demonstrated comparable performance in identifying most of the cephalometric landmarks analyzed in this study, regardless of the population origin of the images. However, certain cephalometric landmarks, such as the Glabella and the Menton L, were not accurately identified by them. Professionals should be aware of this situation and that their performance may be slightly compromised if the origin of the image to be evaluated does not match the origin of the data that trained the software.

## Data Availability

No datasets were generated or analysed during the current study.
